# A dual-branch and dual attention transformer and CNN hybrid network for ultrasound image segmentation

**DOI:** 10.3389/fphys.2024.1432987

**Published:** 2024-09-27

**Authors:** Chong Zhang, Lingtong Wang, Guohui Wei, Zhiyong Kong, Min Qiu

**Affiliations:** ^1^ School of Intelligence and Information Engineering, Shandong University of Traditional Chinese Medicine, Jinan, China; ^2^ Department of Ultrasound Medicine, Affiliated Hospital of Shandong University of Traditional Chinese Medicine, Jinan, China; ^3^ Thyroid Surgery, Jining Medical University, Jining, China

**Keywords:** ultrasound image segmentation, attention mechanism, transformer, convolutional neural network, deep learning

## Abstract

**Introduction:**

Ultrasound imaging has become a crucial tool in medical diagnostics, offering real-time visualization of internal organs and tissues. However, challenges such as low contrast, high noise levels, and variability in image quality hinder accurate interpretation. To enhance the diagnostic accuracy and support treatment decisions, precise segmentation of organs and lesions in ultrasound image is essential. Recently, several deep learning methods, including convolutional neural networks (CNNs) and Transformers, have reached significant milestones in medical image segmentation. Nonetheless, there remains a pressing need for methods capable of seamlessly integrating global context with local fine-grained information, particularly in addressing the unique challenges posed by ultrasound images.

**Methods:**

In this paper, to address these issues, we propose DDTransUNet, a hybrid network combining Transformer and CNN, with a dual-branch encoder and dual attention mechanism for ultrasound image segmentation. DDTransUNet adopts a Swin Transformer branch and a CNN branch to extract global context and local fine-grained information. The dual attention comprising Global Spatial Attention (GSA) and Global Channel Attention (GCA) modules to capture long-range visual dependencies. A novel Cross Attention Fusion (CAF) module effectively fuses feature maps from both branches using cross-attention.

**Results:**

Experiments on three ultrasound image datasets demonstrate that DDTransUNet outperforms previous methods. In the TN3K dataset, DDTransUNet achieves IoU, Dice, HD95 and ACC metrics of 73.82%, 82.31%, 16.98 mm, and 96.94%, respectively. In the BUS-BRA dataset, DDTransUNet achieves 80.75%, 88.23%, 8.12 mm, and 98.00%. In the CAMUS dataset, DDTransUNet achieves 82.51%, 90.33%, 2.82 mm, and 96.87%.

**Discussion:**

These results indicate that our method can provide valuable diagnostic assistance to clinical practitioners.

## 1 Introduction

Medical imaging has assumed a pivotal role in generating visual images of the internal structures of the human body, serving as a vital source of evidence for clinical decision-making over the past few decades (Li J. et al., 2023). There are several widely used medical imaging techniques exist, including magnetic resonance imaging (MRI) (Cabria and Gondra, 2017), computed tomography (CT) (Domingues et al., 2020), radiography, ultrasound (US) imaging (Sloun et al., 2020), and positron emission tomography (PET) (Shamshad et al., 2023). In comparison to these common medical imaging modalities, US imaging stands out for its non-radiative nature, low-cost, real-time capabilities and painless procedures (Jiang et al., 2023). These merits make it particularly suitable for various clinical needs (Huang et al., 2018), such as thyroid nodule detection (Bi et al., 2023), breast tumor screening (Xian et al., 2018), and gynecologic examinations (Drukker et al., 2020).

Medical imaging heavily relies on the pivotal task of medical image segmentation, crucial for the analysis and diagnosis of various diseases. However, even experienced physicians often encounter the formiable challenge of investing considerable manual effort into medical image segmentation, leading to labor-intensive and error-pronen outcomes. This challenge becomes more pronounced in the case of US images, where the relatively low quality and granular noise contribute to the blurriness and inhomogeneity of organ tissues (Chen J. et al., 2020). Consequently, achieving accurate and efficient segmentation of medical ultrasound images has become an indispensable step in clinical applications.

In the last several years, convolutional neural networks (CNNs) have come to be known as dominant forces in the realm of medical image segmentation tasks. The innovative U-Net (Ronneberger et al., 2015), with its efficient encoder-decoder architecture and skip connections, establishes itself as a landmark work demonstrating the effectiveness of such structures within medical image segmentation. Inspired by the remarkable achievement of U-Net, numerous variations have been proposed, including 3D U-Net (Çiçek et al., 2016), UNet++ (Zhou et al., 2018), and Res-UNet (Xiao et al., 2018). These CNN-based networks achieve remarkable performance within the realm of medical image segmentation.

Currently, the revolutionary Transformer architecture, leveraging self-attention as its principal computational component, demonstrates exemplary efficacy in both natural language processing (Devlin et al., 2019) and computer vision (Carion et al., 2020; Wang et al., 2021). The self-attention mechanism within Transformer (Vaswani et al., 2017) exhibits exceptional capabilities in modeling long-range dependencies and capturing global context. The Vision Transformer (ViT) (Dosovitskiy et al., 2020) represents a seminal contribution, demonstrating performance commensurate with alternative CNN-based approaches. Nevertheless, a challenge inherent in pure ViT is its quadratic computational complexity in relation to the input sequence length. Addressing this issue, a hierarchical Swin Transformer (Liu et al., 2021) has been proposed, delivering exceptional results across various visual tasks. Simultaneously, Transformer-based networks have made strides in medical image segmentation. TransUNet (Chen et al., 2021) integrates CNN for feature extraction, feeding the obtained representations into Transformer for global context information learning, with the decoder remaining CNN-based. Swin-Unet (Cao et al., 2023), adopting Swin Transformer as its backbone, stands as the pioneering pure Transformer-based architecture. UNETR (Hatamizadeh et al., 2021), UNETR++ (Shaker et al., 2022) and Swin UNETR (Hatamizadeh et al., 2022) extend Transformer to 3D medical image segmentation and have achieved superior performance.

The Transformer, employing multi-head self-attention to facilitate pairwise entity interactions, excels in capturing long-range dependencies (Naseer et al., 2021). However, when processing long sequences, the Transformer has challenges in effectively capturing local features. Due to extensive interactivity among locations, the Transformer-based model might show excessive blurring or over-smoothing when handing intricate details, resulting in the loss of low-level information. Furthermore, architectures such as TransUNet (Chen et al., 2021) and LeViT-UNet (Xu et al., 2024) are insufficient to fully capture long-range dependency information in features merely by embedding the Transformer at the deepest level. On the other hand, CNNs capture texture features by aggregating local information from contiguous pixels exclusively (Tuli et al., 2021). However, CNNs lack the explicit capability to model global dependencies. Most traditional works based on the Transformer often focus primarily on patch-level spatial attention, limiting the exchange of information among channels to some extent. Relying solely on spatial attention may lead the model to overlook interactions among channels, potentially causing the loss of global contextual information.

The primary impetus for this study arises from the inherent limitations of CNN-based US image segmentation techniques, which often fail to fully encapsulate global contextual information, particularly in scenarios involving complex morphologies, irregular shapes, and multiple lesion targets. Conversely, while the Transformer demonstrates exceptional proficiency in modeling long-range dependencies, it may overlook critical low-level information during processing, which often constitutes the essential local details necessary for achieving precise segmentation. To overcome these limitations and improve mdeical US image segmentation, we propose a U-shaped Transformer and CNN hybrid network named DDTransUNet. This significance of our approach lies in its ability to effectively combine the strengths of both Transformers and CNNs while mitigating their weaknesses. Instead of employing the conventional standard U-shaped encoder framework, DDTransUNet adopts a dual-branch encoder, incorporating a hierarchical Swin Transformer branch and a CNN branch. This dual-branch architecture allows for capturing both global dependencies and local features more comprehensively. To consistently integrate the unique features of the Swin Transformer and CNN branches, a novel Cross Attention Fusion (CAF) module is developed. This module ensures effective feature fusion by facilitating interactions between the branches. In the bottleneck stage, we introduce a global dual attention Transformer block comprising Global Spatial Attention (GSA) module and Global Channel Attention (GCA) module, which further boosts ability of model to capture complete contextual information. In addition, a CNN-based decoder is utilized to complete semantic prediction. In brief, our key contributions are outlined as follows:

(1) We propose DDTransUNet, a hybrid network that adopts a dual-branch encoder architecture, combining a hierarchical Swin Transformer branch and a CNN branch. This unique design allows for the simultaneous extraction of global context from the Swin Transformer branch and local fine-grained features from the CNN branch. This approach addresses the limitations of existing methods by comprehensively capturing both global and local information.
(2) To further enhance the ability to capture complete contextual information, we introduce a global dual attention Transformer block comprising GSA and GCA modules. The GSA concentrates on specific locations or areas within an image, while the GCA emphasizes the interaction of information across different channels. By integrating these two attention mechanisms, the model can consider both important locations in the image and the inter-channel correlations, resulting in a more comprehensive understanding of the image context.
(3) We develop a novel CAF module that leverages a cross-attention mechanism to proficiently fuse features from both the Swin Transformer and CNN branches. This module ensures that the features extracted from each branch are combined in a way that preserves both intricate local details and global contextual information, leading to enhanced spatial information for the semantic features of the skip connection maps.
(4) Our proposed DDTransUNet surpasses current advanced Transformer and CNN-based models on three US image segmentation datasets, which serves as evidence that validates the effectiveness and robustness of our approach.

## 2 Related work

Deep learning techniques have showcased exceptional performance across various domains, notably in precision cancer diagnosis (Tran et al., 2023), accelerated drug development (Le, 2023), and medical image processing. In the specialized domain of medical image processing, two dominant methodologies stand out: one employs CNNs that proficiently analyze image data through a layered feature extraction process; the other leverages the Transformer model, with its formidable ability to handle sequential data.

### 2.1 CNN-based medical image segmentation methods

CNN methods have been extensively researched and implemented within the domain of medical image segmentation. Within this domain, various architectures have emerged to address specific challenges. U-Net (Ronneberger et al., 2015), which comprises a mirrored encoder-decoder architecture with skip connections, has demonstrated considerable success in its application. In addition to U-Net, Attention U-Net (Oktay et al., 2018) incorporates an attention gate mechanism to suppress irrelevant regions while emphasizing salient and useful features. This targeted attention gate mechanism optimizes the capability to focus on pertinent image features, contributing to improved segmentation accuracy. For volume-wise segmentation, both V-Net (Milletari et al., 2016) and 3D U-Net (Çiçek et al., 2016) have achieved substantial success. UNet-2022 (Guo et al., 2023) introduces a non-isomorphic block that combines self-attention and convolution. This block assigns dynamic weights to both spatial positions and channels, resulting in improved performance compared to previous models. U-Net v2 (Peng et al., 2023), as a variant of the U-Net model, refines the fusion of semantic information and fine details, achieving improved segmentation accuracy while maintaining memory and computational efficiency. Zhu et al. (2024) propose a method for brain tumor segmentation in MRI images that integrates a modality information extraction module, a spatial information enhancement module, and a boundary shape correction module to tackle the challenges of multi-modality information utilization, spatial information loss, and boundary information underutilization. Collectively, these diverse CNN-based architectures show the continuous advancements in medical image segmentation techniques.

### 2.2 Transformer-based medical image segmentation methods

Drawing inspiration from the transformative success of the Transformer model in the realm of natural language processing, many researchers have delved into its potential application in computer vision. Vision Transformer, through extensive pre-training and fine-tuning of a pure Transformer, has set the benchmark for image classification datasets. Nevertheless, in the absence of extensive datasets, the performance of ViT diminishes, and the quadratic computational complexity of self-attention in both time and memory exacerbates the challenge. In order to address these obstacle, numerous efficient variants of ViT have been proposed, such as Swin Transformer (Liu et al., 2021), T2T-ViT (Yuan et al., 2021), DeiT (Touvron et al., 2021), Efficient ViT (Liu et al., 2023) and DaViT (Ding et al., 2022). Recently, the success of Transformer in computer vision has spurred the exploration of Transformer-based models in medical image segmentation. TransUNet (Chen et al., 2021) integrates CNN and Transformer in a sequential manner for feature extraction. In a similar vein, UTNet (Gao et al., 2021) innovatively incorporates Transformer blocks, seamlessly weaving them into the encoder and decoder components of the original U-Net. It introduces an efficient self-attention mechanism, resulting in superior segmentation performance without significant computational overhead. Zhu et al. (2023) presents an innovative model for brain tumor segmentation, incorporating a comprehensive approach with three interlinked modules: an advanced semantic segmentation module utilizing an enhanced Swin Transformer, an edge detection module leveraging CNN with an attention mechanism, and a feature fusion module employing graph convolution for effective information propagation. ATTransUNet (Li X. et al., 2023), adopting an innovative token extraction mechanism and a feature enhancement module, achieves outstanding performance. In conclusion, these Transformer-based technique highlight the massive potential of applying the Transformer in the realm of medical image segmentation. AD-DUNet (Qi et al., 2024) utilizes a dual-branch encoder that synergistically integrates the strengths of Transformer and CNN, eliminating the necessity for additional fusion modules. Furthermore, it incorporates efficient Axial Transformer and cascaded dliated convolution modules, delivering superior segmentation performance.

## 3 Methods

### 3.1 Encoder

**FIGURE 1:**
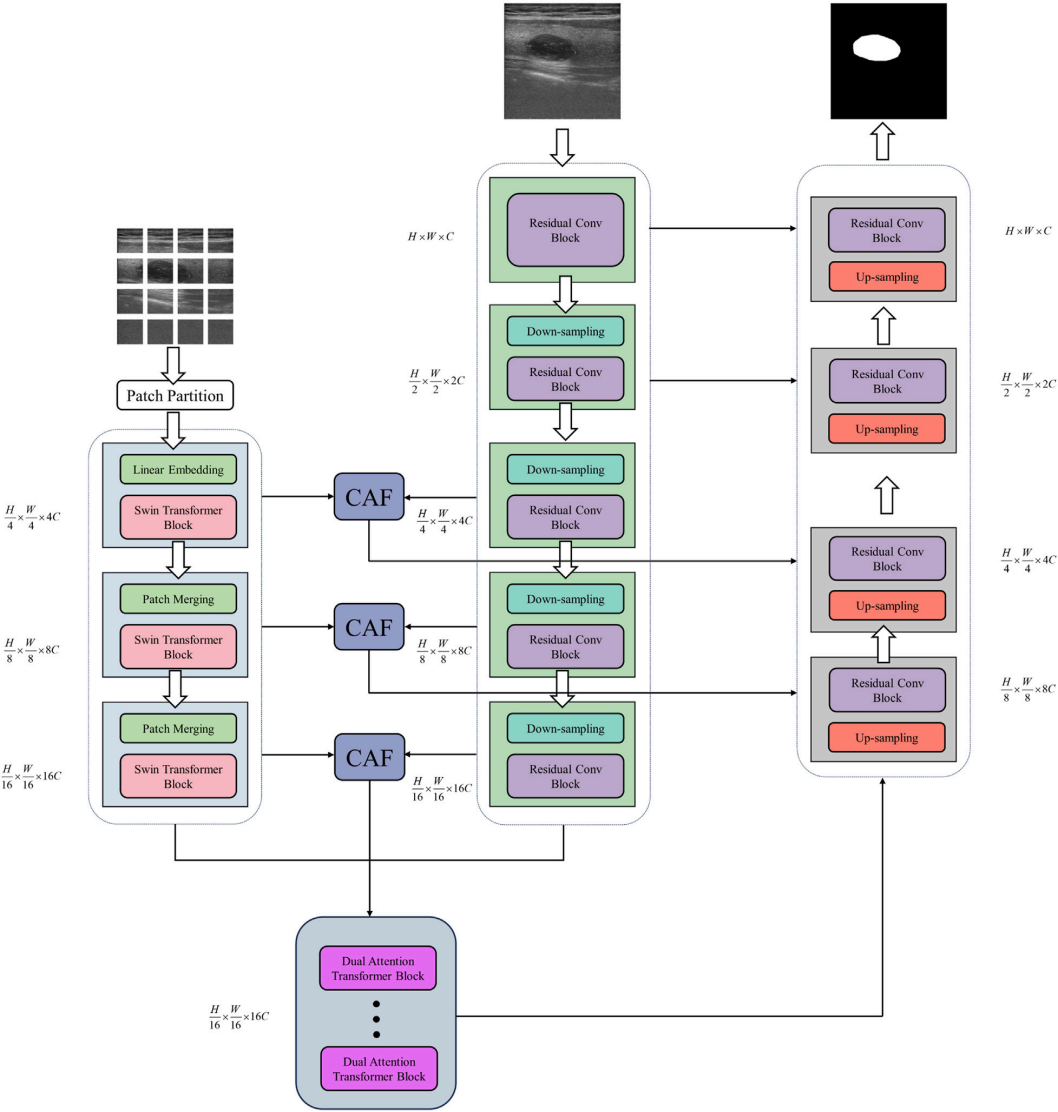
Illustration of the DDTransUNet. The DDTransUNet is a U-shaped structure, adopting a Swin Transformer branch and a CNN branch within its encoder. CAF module is employed to fuse the output feature maps of these two branches. The dual attention Transformer blocks receive the feature maps originating from both branches and the final CAF module.

As shown in Figure 1, the identical ultrasound image is concurrently imported in both the Swin Transformer branch and the CNN branch for simultaneous processing. Subsequently, the dual-branch encoder executes the feature extraction process on the input image, followed by the recovery and reconstruction of the extracted features via the CNN decoder. Ultimately, the network generates a predictive segmentation map. In the context of US image segmentation, a dual-branch encoder combining Swin Transformer and CNN demonstrates significant enhancements. This is chiefly attributed to the self-attention mechanism of Swin Transformer, which adeptly captures global contextual information within the image, thereby effectively modeling long-range visual dependencies. This capability of global modeling is particularly vital in tackling the challenges of low contrast and high noise in US images, as it enables the focus on a broader spectrum of regions, thus enhancing the identification of critical features. The CNN branch is particularly adept at extracting local details and texture features through the use of local receptive fields and weight-sharing mechanisms. In US image segmentation, capturing local details is essential for precise boundary delineation, given that pixels in boundary regions frequently carry important diagnostic information. The dual-branch encoder concurrently processes the Swin Transformer and CNN branches, with each branch dedicated to extracting global contextual information and local detail features, respectively. This parallel processing harnesses the strengths of both networks in unison, thereby augmenting the comprehensiveness and precision of feature extraction. The features extracted from both branches, possessing identical resolution, are seamlessly integrated into our innovative Cross Attention Fusion Module, where cross-attention is applied to fuse both local and global contextual features.

Specifically, the first two phases of the CNN branch are processed independently, while the last three stages are jointly processed by the CNN branch and the Swin Transformer branch. During the joint processing phase, the features derived from CNN and Swin Transformer can be fused across multiple levels. This hierarchical fusion maximizes the strengths of both, resulting in a richer and more comprehensive feature representation. Particularly when handing complex images, this fusion strategy markedly anhances model performance.

#### 3.1.1 Swin Transformer branch

**FIGURE 2:**
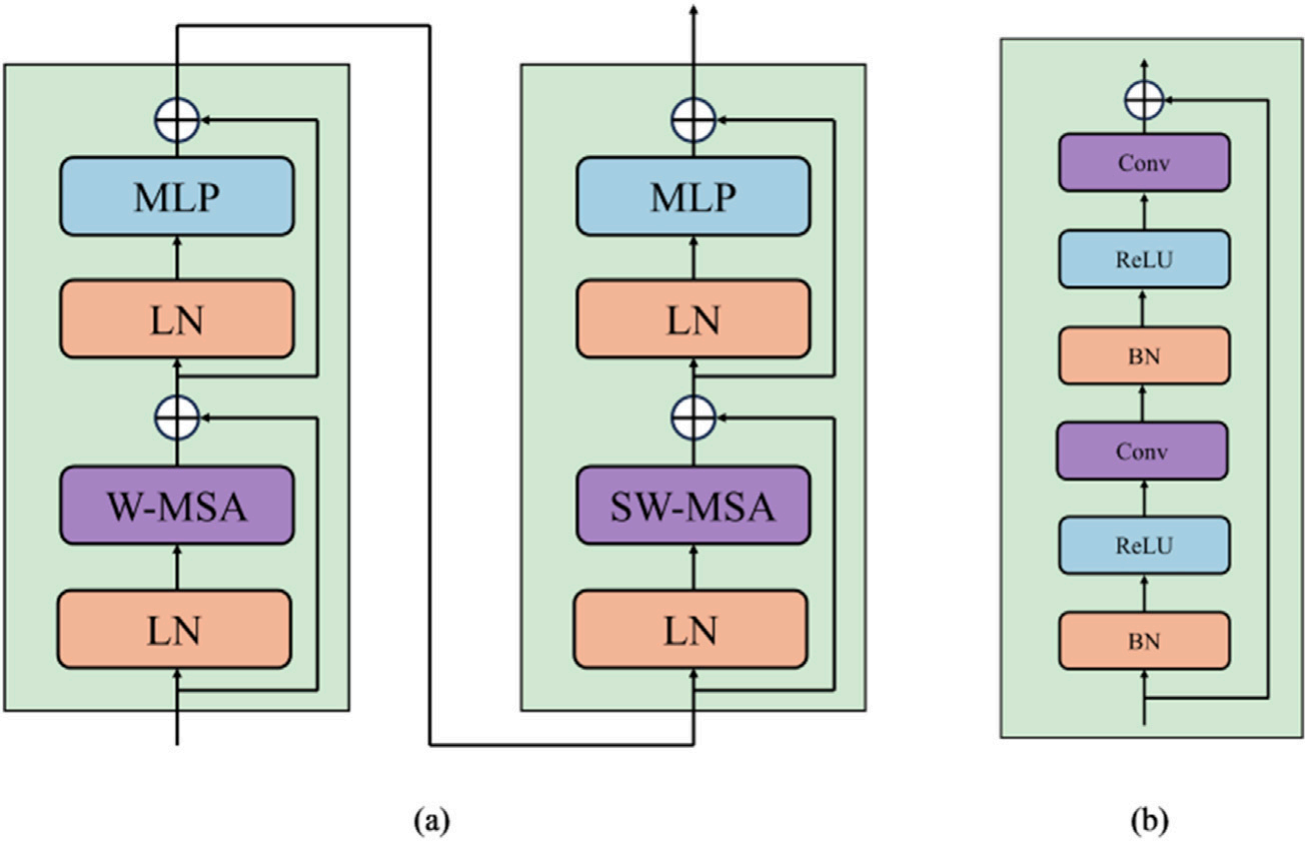
**(A)** The structure of Swin Transformer block. **(B)** The structure of Residual Convolution block.

Conventional Vision Transformer architecture employs global self-attention, leading to a quadratic computational complexity. Contrastly, the Swin Transformer computes its self-attention within a delimited region, focusing attention within a specified window. As shown in Figure 2A, each Swin Transformer block is composed of a window multi-head self-attention (W-MSA) or shifted window multi-head self-attention (SW-MSA) module. The Formulas 1–4 utilized for calculating the two-layer features of the continuous output generated by Swin Transformer blocks is presented below:

$${\hat{z}}^{l}=W-MSA\left(\mathrm{LN}\left(z^{l-1}\right)\right)+z^{l-1}$$ (1)

$$z^{l}=MLP\left(\mathrm{LN}\left({\hat{z}}^{l}\right)\right)+{\hat{z}}^{l}$$ (2)

$${\hat{z}}^{l+1}=SW-MSA\left(\mathrm{LN}\left(z^{l}\right)\right)+z^{l}$$ (3)

$$z^{l+1}=MLP\left(\mathrm{LN}\left({\hat{z}}^{l+1}\right)\right)+{\hat{z}}^{l+1}$$ (4)

where MLP denotes a Multi Layer Perceptron (Taud and Mas, 2018) layer, LN denotes a LayerNorm (Ba et al., 2016) layer, ${\hat{z}}^{l}$ and $z^{l}$ denote the extracted features from the W-MSA module and the MLP layer for block *l*, ${\hat{z}}^{l+1}$ and $z^{l+1}$ denote the extracted features from the SW-MSA module and the MLP layer for the subsequent block *l+1*, respectively.

The input of Swin Transformer branch is an ultrasound image $x\in R^{H\times W\times C}$ , where the height is *H*, the width is *W*, and the total number of channels is C. As depicted in Figure 2, the ultrasound image is initiallt employed the overlapping patch embedding approach (Zhou et al., 2023), leveraging successive convolutional layers with small convolutional kernels to the same dimensions as the third stage of the CNN branch. For this way has the ability to encode spatial information at the pixel level, it more completely retains local neighborhood features within the image. Conversely, non-overlapping patch embedding (Dosovitskiy et al., 2020) may result in partial or complete loss of critical local correlation information, as they fail to account for overlap between patches. Specifically, there are two sets of operations, each operation is structured with a 3×3 convolution, utilizing a stride of 2, succeeded by another 3×3 convolution employing a stride of 1, along with a GELU activation and a normalization layer. The resulting patches are finally fed into the Swin Transformer, structured into three stages. In order to achieve a hierarchical representation, the first two output features of the Swin Transformer blocks go through downsampling layers. This process reduces feature resolution and increases dimensionality. In our approach, we replace the original patch merging operation with a simple 3×3 convolution with a stride of 2 for downsampling.

#### 3.1.2 CNN branch

The CNN branch comprises five groups of convolutional blocks. CNNs predominantly focus on extracting fundamental, low-level features like edges and textures during the initial phases of image processing, especially within the first few layers. These features serve as the essential building blocks for subsequent complex pattern recognition tasks. Independently processing the initial two stages of the CNN guarantees that these foundational features are thoroughly and accurately extracted, thus establishing a robust basis for subsequent advanced feature fusion. As the depth of the network layers increases, CNNs progressively extract more sophisticated and abstract features, whereas the Swin Transformer excels in capturing global dependencies and long-range features within images through its distinctive self-attention mechanism. The joint processing of the latter three stages of CNN with the Swin Transformer fully leverages their complementary strengths, enabling the model to accurately capture local details while comprehending the global structure. Moreover, while Swin Transformer excels with large-scale datasets, it may encounter difficulties with small-sclae datastes due to the absence of the inductive bias that is inherent in CNNs. This limitation can be alleviated by independently processing the initial two stages of the CNN and integrating the Swin Transformer in the latter three stages. The inductive bias inherent in CNNs facilitates superior performance on small-sclae datastes, whereas the Swin Transformer augments the capacity to capture intricate patterns and enhances generalization.

As described in Figure 2B, each block integrates convolutional layers, batch normalization (Ioffe and Szegedy, 2015) layers, and ReLU activation (Agarap, 2018). Utilizing a 3×3 convolution, along with a stride of 2 and a padding value of 1, we ensure an effective feature extraction process. Additionally, the downsampling is performed in a similar manner to the Swin Transformer branch.

#### 3.1.3 Cross attention fusion module

In US images, marked heterogeneity in the size and number of lesions is observed, a feature that presented a formidable challenge to the precise identification of all target lesions. To effectively integrate global and local features, thereby augmenting the recognition of pathological tissues, such as tumors, in ultrasound images and ensuring the model meticulously captures both the macroscopic structure and the nuanced details of the diseased tissue, we propose a Cross Attention Fusion (CAF) module. The Swin Transformer branch captures long-distance visual dependencies with its self-attention mechanism, resulting in feature maps characterized by comprehensive global feature representations. On the other hand, the CNN branch extracts more local feature representations through its exceptional spatial perception capability. The CAF module achieves efficient and effective fusion of local and global featurs by employing a cross attention operation on the extracted feature representations derived from both the Swin Transformer branch and the CNN branch. This cross attention operation learns from the different feature representations of these two branches, enhancing the expressive power of the final feature map.

**FIGURE 3:**
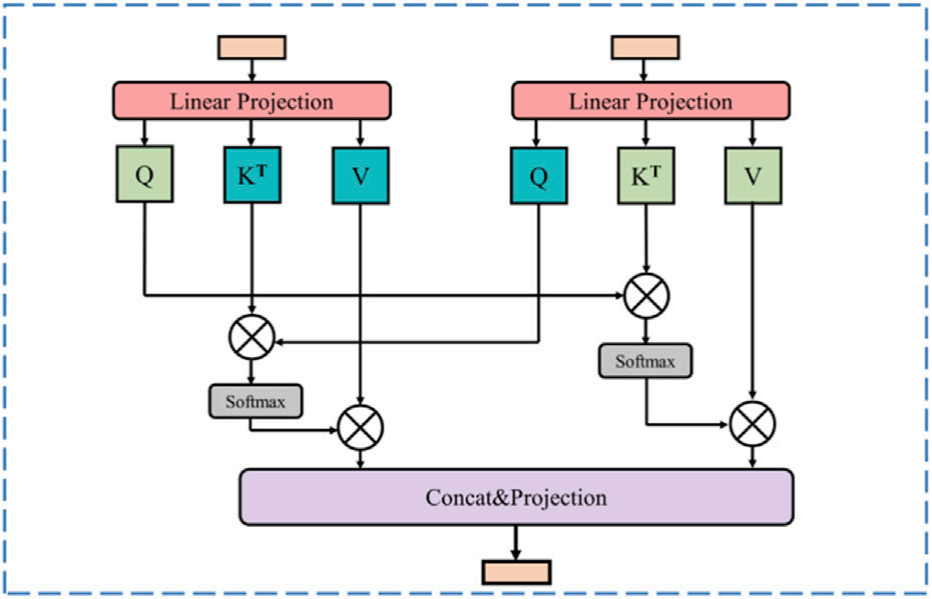
Illustration of cross attention fusion module (CAF). At the same stage, the feature maps generating from the Swin Transformer branch and the CNN branch are fused through the cross-attention operation.

As shown in Figure 3, for a given stage *i* (*i* = 1, 2, 3), the Swin Transformer branch produces a map represented as *F* ^*i*^, while the CNN branch generates a map denoted as *G* ^*i*^. Firstly, the cross attention between *F* ^*i*^ and *G* ^*i*^ in Formulas 5, 6 can be computed as follows:

$$CrossAttention\left(Q_{F^{i}},K_{G^{i}},V_{G^{i}}\right)=softmax\left(\frac{Q_{F^{i}}{K_{G^{i}}}^{T}}{\sqrt{d}}\right)V_{G^{i}}$$ (5)

$$CrossAttention\left(Q_{G^{i}},K_{F^{i}},V_{F^{i}}\right)=softmax\left(\frac{Q_{G^{i}}{K_{F^{i}}}^{T}}{\sqrt{d}}\right)V_{F^{i}}$$ (6)

Where $Q_{F^{i}}$ , $K_{F^{i}}$ , $V_{F^{i}}$ indicate the query, key, value matrices of *F* ^*i*^, and $Q_{G^{i}}$ , $K_{G^{i}}$ , $V_{G^{i}}$ stand for the qurey, key, value matrices of *G* ^*i*^. Then, the two maps *F* ^*i*^ and *G* ^*i*^ are merged through concatenation and subsequently performed a linear projection, yielding the desired final output.

With the CAF module, the high-level global contextual feature representations acquired from the Swin Transformer branch and the local fine-grained feature representations derived from the CNN branch are fully utilized, enabling the feature map to incorporate both long-range context dependencies and fine-grained details.

### 3.2 Bottleneck

**FIGURE 4:**
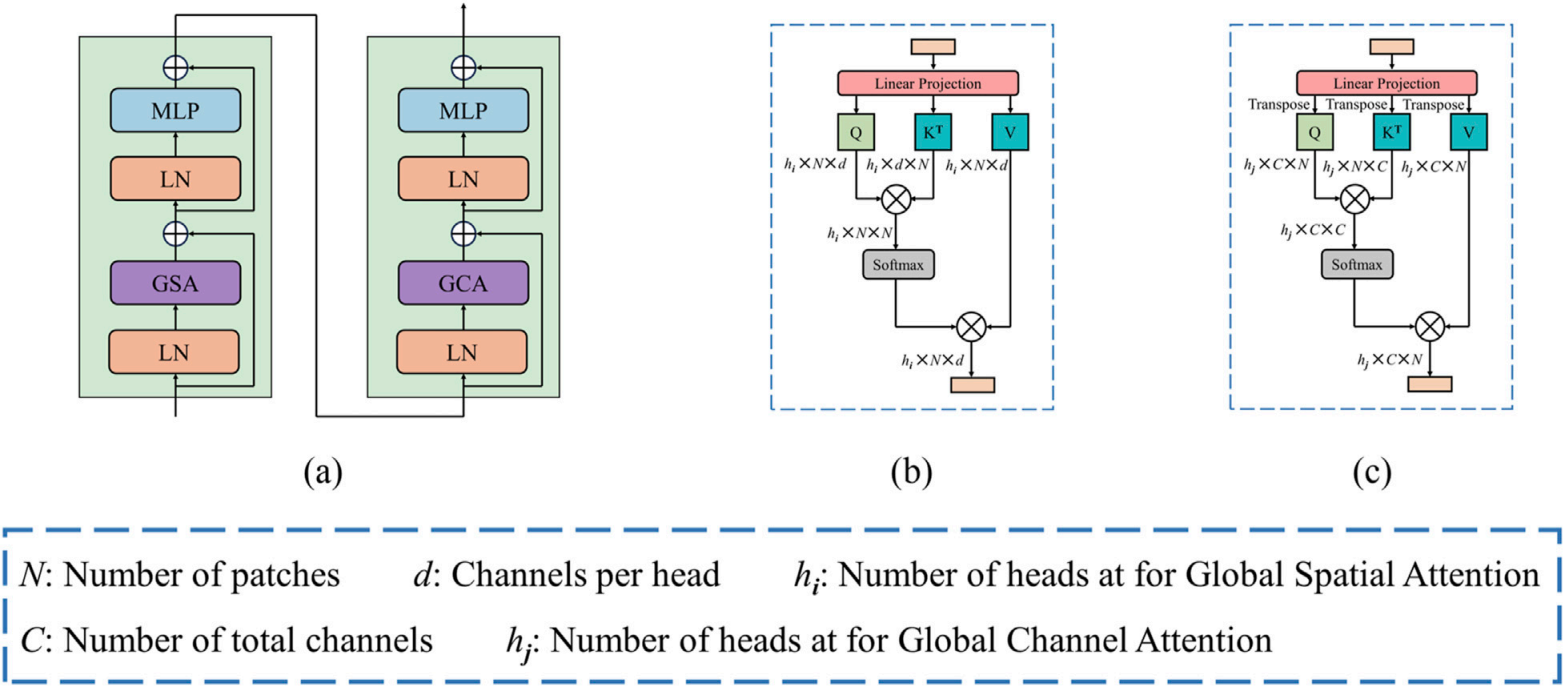
**(A)** Architecture of dual attention block. **(B)** Global spatial attention. **(C)** Global channel attention.

US images often blur the boundaries between diseased and adjacent tissues due to the common issue of insufficient contrast. To enhance the relevance of image features and bolster the discriminative capability of the model, as illustrated in Figure 4A, we propose a global dual attention Transformer block, which comprises of the Global Spatial Attention (GSA) module and the Global Channel Attention (GCA) module. This block effectively captures long-range visual feature information, enabling the model to concentrate on the structural details of the lesion region during feature extraction, hence improving the discrimination between lesion tissue and surrounding tissues.

Following the concatenation of features obtained from the Swin Transformer branch, the CNN branch, and the final CAF module, the combined features are initially processed through a patch embedding layer. Subsequently, we apply our global dual attention blocks to these encoder tokens to modeling long-range visual dependencies.

#### 3.2.1 Global spatial attention

The GSA emphasizes the spatial positioning within an image, enabling the model to more effectively concentrate on regions of interest by assigning varying weights to individual pixels. This mechanism is especially crucial in US images, which frequently contain noise, artifacts, and srtuctural complexities. By leveraging GSA, the model can more accurately delineate the boundaries and shape of the target region, thus enhancing segmentation accuracy and minimizing the likelihood of false positives or missed segmentations.

Illustrated in Figure 4B, the GSA module operates on a provided feature map $X\in R^{N\times d}$ , the Formula 7 for GSA can be represented by:

$$GSA\left(Q_{S},K_{S},V_{S}\;\right)=softmax\left(\frac{Q_{S}{K_{S}}^{T}}{\sqrt{d_{s}}}\right)V_{S}$$ (7)

where $Q_{S}\in R^{N\times d}$ , $K_{S}\in R^{N\times d}$ , $V_{S}\in R^{N\times d}$ denote the projected qurey, key and value matrices, respectively, *N* signifies the number of patches, *d* represents the dimension per head, and $\frac{1}{\sqrt{d_{S}}}$ serves as the scaling factor. By leveraging this mechanism, the model can dynamically focus on information across different spatial positions, enabling it to capture and integrate information from various representation subspaces simultaneously.

#### 3.2.2 Global channel attention

By allocating distinct weights to different channels, the GCA modulates the focus of the model on various features. In US images, each channel typically represents specific features, such as edges, textures, or higher-order semantic information. Through GCA, the model gains the flexibility to selectively focus on the features most relevant to the segmentation task, thereby enhancing the precision of the segmentation outcomes.

Figure 4C illustrates the GCA, it can be computed from the transpose of the patch-level tokens. Similarly, given a token map $X\in R^{N\times C}$ , the Formula 8 for GCA can be defined as:

$$GCA\left(Q_{C},K_{C},V_{C}\;\right)=softmax\left(\frac{{Q_{C}}^{T}K_{C}}{\sqrt{c}}\right){V_{C}}^{T}$$ (8)

where $Q_{C}\in R^{N\times C}$ , $K_{C}\in R^{N\times C}$ , $V_{C}\in R^{N\times C}$ stand for the projected qurey, key and value matrices respectively, *N* represents the total count of patches, *C* denotes the dimension per patch, and $\frac{1}{\sqrt{c}}$ serves as the scaling factor. By utilizing the GCA, image-level tokens can effectively interact across different channels, enabling the model to integrate information and more long-range dependencies across the entire image representation.

### 3.3 Decoder

The decoder mainly consists of four stages, with each stage containing an up-sampling layer that utilizes deconvolution for enhancing the spatial resolution of the feature maps. After that, a Residual Convolutional block follows in each decoder stage. As shown in Figure 1, the output from the global dual attention blocks serves as initial input for the decoder. During the first two stages of the decoder, the features from the lower resolution are up-sampled and then concatenated with the features from the Cross Attention Fusion module. In the final two stages of the decoder, the extracted features are concatenated with those originating from the CNN branch within the encoder.

### 3.4 Complexity analysis

We analyze the computational complexity of the problem. For a fearure map with height *h* and width *w*, and containing *C* channels, the Formula 9 for computational complexity of processing using the window-based self-attention mechanism can be delineated as follows, given a window size of M:

$$O\left(W-MSA\right)=4hwC^{2}+2M^{2}hwC$$ (9)

The Formulas 10, 11 for computational complexity of GSA and GCA is as follows:

$$O\left(GSA\right)=4hwC^{2}+2{\left(hw\right)}^{2}C$$ (10)

$$O\left(GCA\right)=2hwC^{2}+2{\left(hw\right)}^{2}C$$ (11)

In the bottleneck stage, the computational burden is alleviated as the feature map undergoes successive spatial downsampling processes, leading to a relatively low computational complexity at this stage.

## 4 Experiments

### 4.1 Datasets

In this paper, we use three widely accessible ultrasound image datasets to assess the effectiveness of our method, including the TN3K dataset (Gong et al., 2021), BUS-BRA dataset (Gómez-Flores et al., 2024), and CAMUS dataset (Leclerc et al., 2019). The TN3K comprises 3,493 ultrasound images of thyroid nodules, originating from 2,421 patients. These are further categorized into 2,879 training samples and 614 test samples. Similarly, the BUS-BRA dataset contains 1,875 breast ultrasound images, sourced from 1,064 female patients. These are divided into 1,500 training samples and 375 test samples. Lastly, the CAMUS dataset consists of 2,000 ultrasound images. Which are partitioned into 1,800 training samples and 200 test samples.

### 4.2 Evaluation metrics

To quantitatively evaluate the segmentaion capabilities of our proposed DDTransUNet, we employ Intersection Over Union (IoU), Dice Coefficient (Dice), 95% Hausdorff Distance (HD95) and Accuracy (ACC) as our evaluation metrics. The formulas are shown in Equations 12–15.

$$IoU=\frac{TP}{TP+FP+FN}$$ (12)

$$Dice=\frac{2\times TP}{TP+FP+TP+FN}$$ (13)

$$HD\left(X,Y\right)=\max\left\{\max_{x\in X}\min_{y\in Y}d\left(x,y\right),\max_{y\in Y}\min_{x\in X}d\left(x,y\right)\right\}$$ (14)

$$ACC=\frac{TP+TN}{TP+TN+FP+FN}$$ (15)

where TP represents true positive, TN represents true negative, FP represents false positive, and FN represents false negative, X and Y represent ground truth pixels and predicted pixels.

### 4.3 Implementation details

The loss function used by us is binary cross entropy loss and dice loss, and can be formulated as Equation 16:

$$L=0.5L_{BCE}+L_{Dice}$$ (16)

The model is constructed using the open-source framework PyTorch and trained from scratch for 200 epochs on an NVIDIA V100 GPU. We use the Adam optimizer along with a cosine-based learning rate scheduler, ensuring a minimum learning rate of 1e-5. Additionally, the batch size remains consistent and set to 8. The input images are preprocessed to have a resolution of 256 × 256. The initial learing rate is 1e-4 and momentum of 0.9.

### 4.4 Comparison with state-of-the-art methods

We have conducted comprehensive quantitative and qualitative comparsions with various segmentation techniques, encompassing both CNN-based frameworks and Transformer-based methodologies. The compared methods include U-Net (Ronneberger et al., 2015), UNet++ (Zhou et al., 2018), ResUNet++ (Jha et al., 2019), UNeXt (Valanarasu and Patel, 2022), MTUNet (Wang et al., 2022), TransUNet (Chen et al., 2021), MISSFormer (Huang et al., 2023) and Swin-Unet (Cao et al., 2023).

**FIGURE 5:**
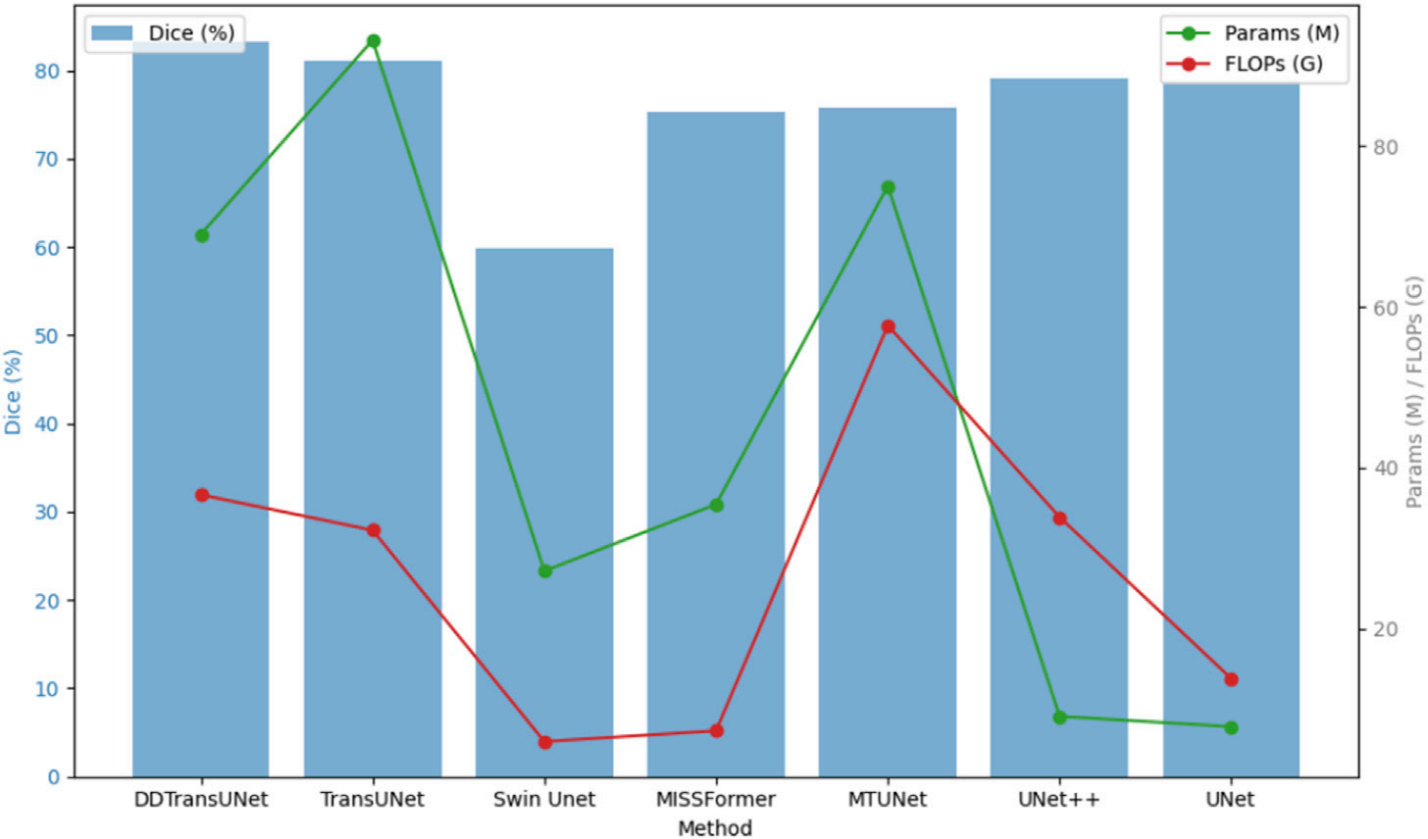
Dice coefficient on the TN3K dataset for various models.

To asses the performance of DDTransUNet against baseline models in US image segmentation, we specifically examine the interaction between the number of parameters, the number of floating-point operations per second (FLOPs) as primary metics for evaluating computational complexity, and the Dice coefficient as the fundamental standard for segmentation accuracy. This multidimensional analytical framework is designed to thoroughly evaluate the capability of model to balance high-precision segmentation outcomes with computational efficiency and resource consumption. Specifically, the number of parameters, as a direct indicator of model complexity, is closely associated with memory usage and inference loading speed. Conversely, FLOPs quantify the computation needed for a model to perform a forward pass, serving as a crucial indicator of processing speed. As shown in Figure 5, our DDTransUNet exhibits significant advantages over conteporary mainstream hybrid network combining CNN and Transformer. Our model not only achieves a substantial improvement in performance, as evidenced by a significant increase in the Dice coefficient, but more importantly, it successfully reduces the required number of model parameters and computational complexity. This dual adnvantage highlights the efficiency of DDTransUNet in extracting and utilizing feature information, while also enhancing its suitability for deployment in resource-constrained real-world clinical environments.

As illustrated in Tables 1–3, our proposed network consistently attains superior performance across all three datasets. Specifically, DDTransUNet achieves an IoU of 73.82%, 80.75% and 82.51%, Dice of 82.31%, 88.23%, and 90.33%, HD95 of 16.98, 8.12, and 2.82 mm, and ACC of 96.94%, 98.00%, and 96.87% on the TN3K, BUS-BRA and CAMUS dataset, respectively. In terms of IoU, DDTransUNet has an improvement of 1.26%, 0.81% and 1.33%, respectively, when compared with the second-best model. In terms of Dice, DDTransUNet has achieved a 1.27%, 0.66% and 0.79% improvement in comparison to the second-best model. In terms of HD95, DDTransUNet has achieved a 1.22, 0.99 and 0.07 mm improvement, respectively, compared with the second-best model. In terms of ACC, DDTransUNet has an improvement of 0.05%, 0.04% and 0.27%, respectively, when compared with the second-best model. Furthermore, the statistical analysis indicates that the p-values in all instances consistently fall below the significance level threshold of 0.05. This result strongly suggests that the methodology proposed in this study achieves a significant improvement in performance compared to the current state-of-the-art methods of its kind.

**TABLE 1:** Quantitative results of different methods on TN3K dataset.

Methods	IoU (%)	Dice (%)	HD95 (mm)	ACC (%)	p-value
U-Net	69.42	78.75	22.67	96.40	1.46e-7
UNet++	70.36	79.15	24.79	96.56	1.46e-6
ResUNet++	71.76	80.84	18.99	96.73	9.65e-3
UNeXt	65.40	75.68	25.32	96.15	1.39e-23
MTUNet	65.82	75.83	23.46	95.83	7.42e-17
TransUnet (ViT)	52.60	64.30	36.83	94.12	3.56e-66
TransUnet (R50-ViT)	72.56	81.04	18.20	96.89	4.21e-2
MISSFormer	65.27	75.23	20.47	95.77	4.53e-19
Swin-Unet	47.94	59.94	34.16	93.17	9.92e-83
DDTransUNet (ours)	**73.82**	**82.31**	**16.98**	**96.94**	**-**

The best results are marked with bold text.

**TABLE 2:** Quantitative results of different methods on BUS-BRA dataset.

Methods	IoU (%)	Dice (%)	HD95 (mm)	ACC (%)	p-value
U-Net	79.33	87.00	10.10	97.94	1.58e-2
UNet++	79.02	86.74	11.69	97.90	7.38e-3
ResUNet++	79.30	86.90	10.41	97.88	3.47e-2
UNeXt	77.31	85.28	11.31	97.80	1.05e-5
MTUNet	79.74	87.53	9.30	97.96	2.31e-1
TransUnet (ViT)	67.70	78.35	20.33	96.78	6.45e-23
TransUnet (R50-ViT)	79.94	87.08	9.11	97.94	4.94e-2
MISSFormer	75.85	84.16	11.07	97.46	2.28e-8
Swin-Unet	63.02	73.87	20.27	96.15	1.12e-32
DDTransUNet (ours)	**80.75**	**88.23**	**8.12**	**98.00**	**-**

The best results are marked with bold text.

**TABLE 3:** Quantitative results of different methods on CAMUS dataset.

Methods	IoU (%)	Dice (%)	HD95 (mm)	ACC (%)	p-value
U-Net	81.18	89.54	2.96	96.60	7.08e-6
UNet++	80.54	89.13	3.18	96.42	3.35e-12
ResUNet++	80.80	89.31	2.89	96.50	6.11e-9
UNeXt	75.17	85.73	4.65	95.19	2.94e-52
MTUNet	78.93	88.15	3.64	96.09	1.01e-26
TransUnet (ViT)	73.89	84.83	5.37	94.99	3.35e-52
TransUnet (R50-ViT)	79.86	88.72	3.26	96.34	3.79e-18
MISSFormer	77.87	87.46	3.44	95.85	2.29e-36
Swin-Unet	71.21	83.05	5.96	94.12	2.06e-74
DDTransUNet (ours)	**82.51**	**90.33**	**2.82**	**96.87**	**-**

The best results are marked with bold text.

**FIGURE 6:**
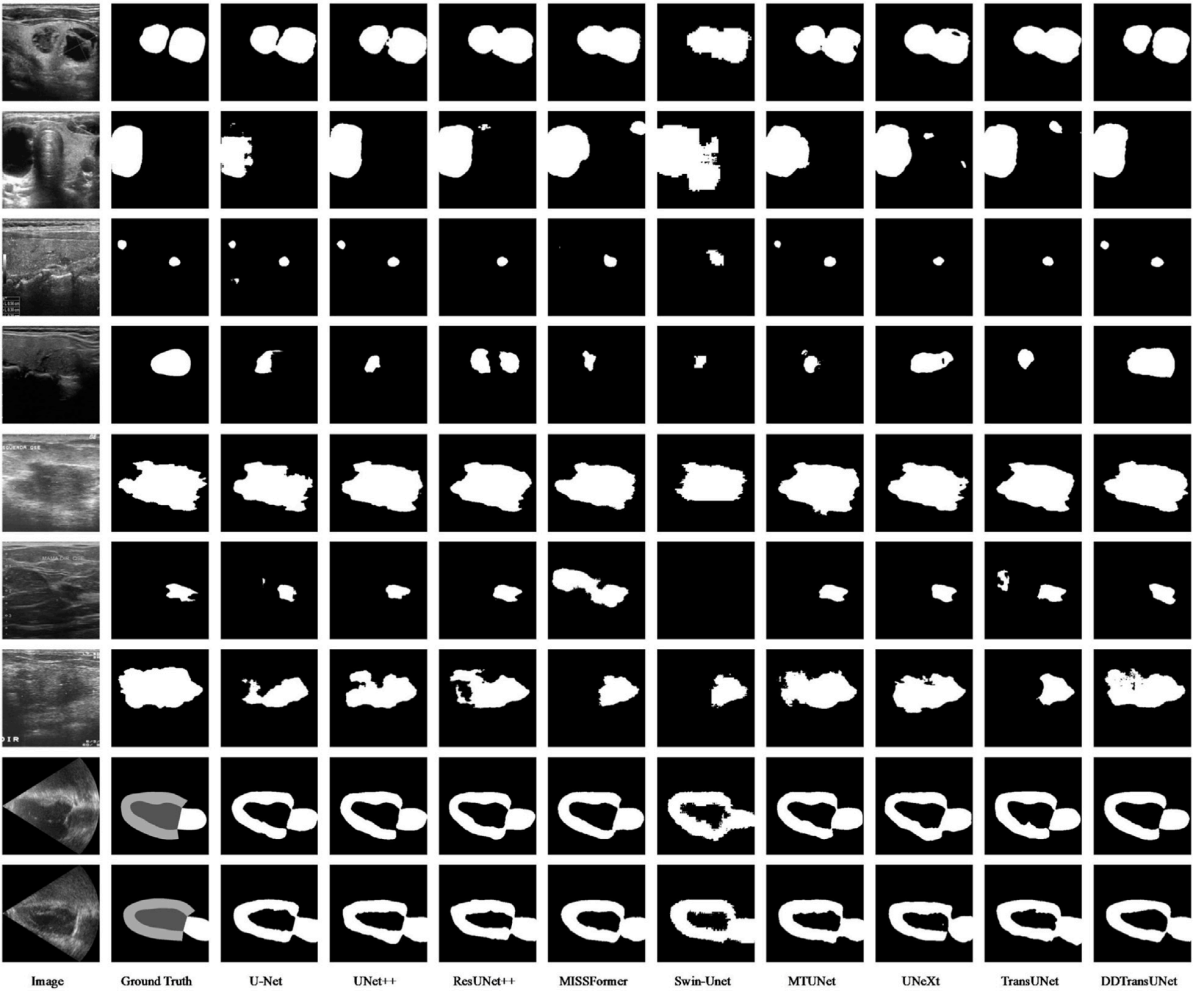
Qualitative segmentation results on the TN3K, BUS-BRA and CAMUS dataset.

Additionally, we carry out a qualitative analysis, evaluating and comtrasting the outcomes of segmentation achieved by various methods across three datasets. The visual results are exhibited in Figure 6. The segmentation of ultrasound images presents challenges arising from their low contrast, non-uniform features, and ambiguous object boundaries. Current methods encounter difficulties in discriminating the target from the background, posing a challenge in accurately delineating the complete lesion tissue. In particular, when dealing with multiple thyroid nodules in the TN3K dataset, in the first and second rows, certain models mistakenly identify the non-lesion regions as part of the lesion. In contrast, our DDTransUNet excels in accurately identifying multiple nodules due to the synergy of GSA and GCA. This dual attention enables the model to simultaneously focus on spatial and channel dimensions, thereby providing a more holistic comprehension of the image content. When tasked with identifying multiple thyroid nodules, the DDTransUNet employs spatial attention to concentrate on the locations and morphologies of the nodules, while channel attention accentuates features pertinent to nodule identification. This synergy boosts the ability of model to accurately identify multiple nodules in an image, thereby mitigating the risks of misclassification and omission. Furthermore, the majority of models in the third and fourth rows fail to precisely delineate all nodules present within the depicted image. Within the BUS-BRA dataset, in the fifth row, the boundaries encapsulating the lesion regions exhibit intricate characteristics, thereby rendering the segmentation outcomes of some models unsatisfactory and impeding their precision in delineating the boundaries. Simultaneously, in the seventh row, the previous methods have conspicuous constraints in achieving detailed extraction of the lesion regions. It can be observed that our DDTransUNet has the most favorable segmentation results in all three datasets. This is can be attributed to the dual-branch encoder of DDTransUNet to capture features which contain both local and global semantic information. The local feature information extracted by the CNN branch substantially amplifies the boundary detail, whereas the Swin Transformer branch fosters the integrity and continuity of the segmented regions, with its broader field-of-view focus.

**FIGURE 7:**
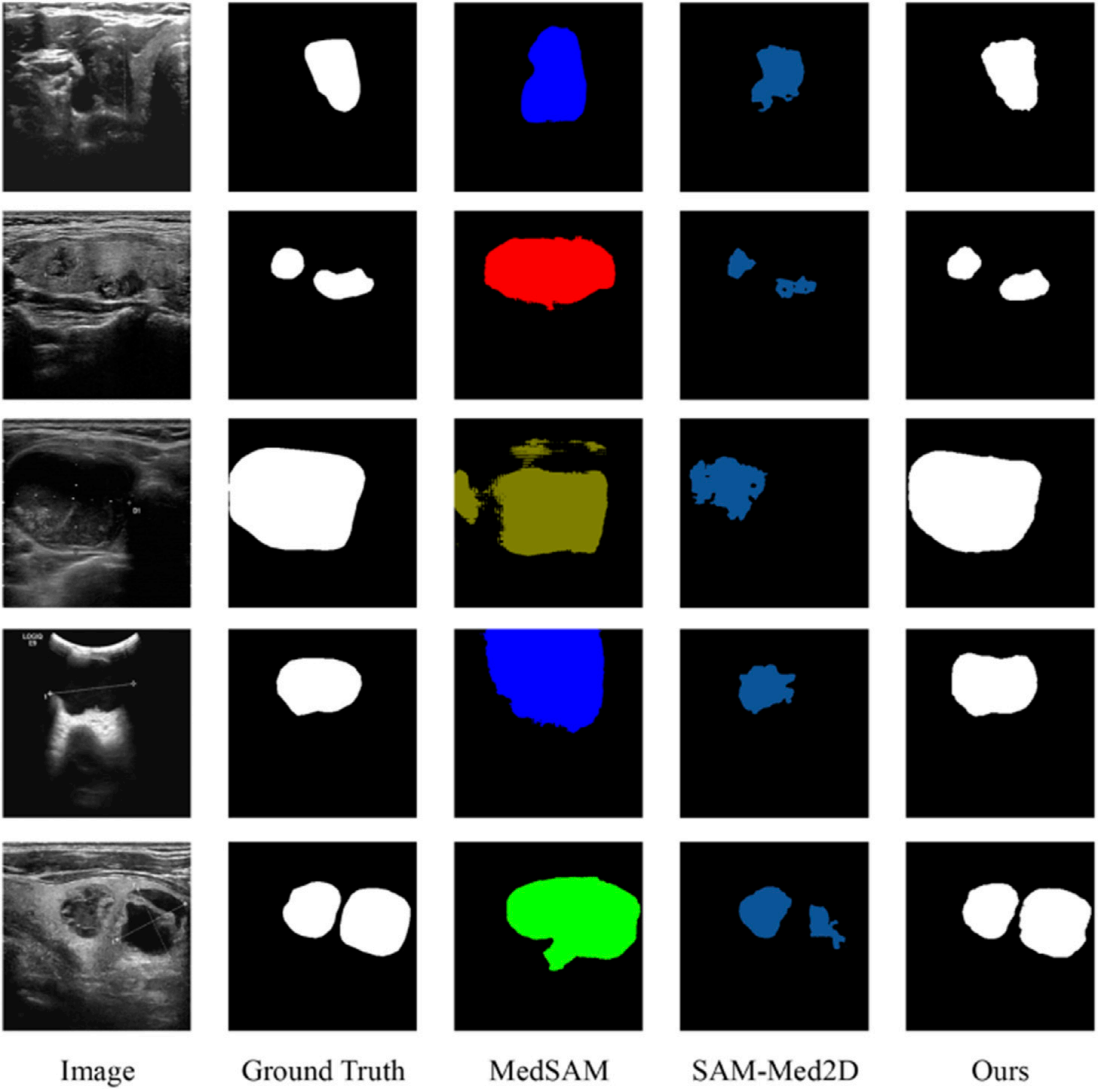
Visualization segmentation results with medical generalized segmentation models.

Recently, numerous advanced segmentation models, such as SAM (Kirillov et al., 2023) and U2seg (Niu et al., 2023), have gained significant attention for their generalizability. These generalized segmentation techniques have been innovatively applied to medical image segmentation, with specific examples including MedSAM (Ma et al., 2024), UniSeg (Ye et al., 2023), and SAM-Med2D (Cheng et al., 2023), demonstrating considerable potential for cross-domain applications. We have performed an exhaustive comparative analysis of our proposed DDTransUNet against MedSAM and SAM-Med2D, with visualization results in Figure 7. The results indicate that while these generalized segmentation models designed for medical applications exhibit some value in medical image processing, they struggle with accurately defining segmentation boundaries in US images, leading to mis-segmentation. In contrast, our network shows superior performance, accurately capturing and defining segmentation boundaries and effectively recognizing multi-target segmentation scenarios. This significant improvement its primarily attributed to the dual-branch encoder, which facilitates the efficient complementation and fusion of global and local information, thereby laying a solid foundation for improved segmentation accuracy.

### 4.5 Ablation study

#### 4.5.1 Effectiveness of dual-branch encoder

We first conduct an experiment to validate the efficacy of the proposed dual-branch encoder design, comparing scenarios where only the Swin Transformer or CNN is employed as the encoder. The results are shown in Table 4. The inclusion of our dual-branch encoder, when compared with using only the Swin Transformer encoder, results in improvements of 6.87% and 5.18% in IoU and Dice on the TN3K dataset, and 3.41% and 2.74% on BUS-BRA dataset, respectively. Simultaneously, in comparsion to utilizing only the CNN encoder, our dual-branch encoder also have an improvements of 0.50% and 0.14% in IoU and Dice on the TN3K dataset, and 0.15% and 0.17% on the BUS-BRA dataset, respectively. In conclusion, the introduction of the dual-branch encoder results in a significant advancement in performance in comparsion to the single-branch scenario.

**TABLE 4:** Ablation study on TN3K and BUS-BRA datasets.

Methods					TN3K		BUS-BRA	
Swin encoder	CNN encoder	CAF	GSA	GCA	IoU (%)	Dice (%)	IoU (%)	Dice (%)
✓					63.05	73.71	76.07	84.43
	✓				69.42	78.75	79.33	87.00
✓	✓				69.92	78.89	79.48	87.17
✓	✓	✓			70.89	80.11	79.65	87.32
✓	✓	✓	✓		73.11	81.72	80.51	88.06
✓	✓	✓		✓	73.40	82.05	80.28	87.74
✓	✓	✓	✓	✓	**73.82**	**82.31**	**80.75**	**88.23**

The best results are marked with bold text.

#### 4.5.2 Effectiveness of CAF module

Then, to assess the effectiveness of the novel CAF module, we substitute the original CAF module with a straightforward operation. It can be observed that in Table 4, our network achieve IoU and Dice improvements of 0.97% and 1.22% on the TN3K dataset and 0.17% and 0.15% on the BUS-BRA dataset, following the incorporation of the CAF module. It can be concluded that the CAF module facilitates more efficient feature aggregation from different branches. As a result, it contributes superior segmentation performance.

#### 4.5.3 Effectiveness of global dual attention

We finally evaluate the effectiveness of the Global Dual Attention. We structure our experiments into three distinct configurations: firstly, by applying solely the GSA; secondly, by integrating only the GCA; and thirdly, by combining both GSA and GCA. In Table 4, we can see that employing the global dual attention leads to improvements of 0.71% and 0.42% in terms of IoU and 0.59% and 0.26% in terms of Dice on the TN3K dataset, 0.24% and 0.47% in terms of IoU and 0.17% and 0.49% in terms of Dice on the BUS-BRA dataset, respectively, compared to scenarios where only the GSA or GCA is utilized. One can deduce that the implementation of the global dual attention mechanism significantly enhances the capabilities of medical ultrasound image segmentation.

## 5 Discussion

In the field of medical US imaging, it is widely recognized that the quality of imaging is relatively constrained, with low contrast between specific tissues or lesions and their surrounding background tissues, leading to challenges in visual identification. In this study, we propose DDTransUNet, a hybrid network that combines Transformer and CNN architectures, with a dual-branch encoder for feature extraction and dual attention mechanisms to model long-range dependencies for medical US image segmentation.

Compared to the Transformer-based, MLP-based and CNN-based networks, we observe that the CNN-based networks outperform the former in terms of segmentation effectiveness. Following a thorough analysis, this phenomenon primarily stems from the relatively small dataset size, which limits the advantages of Transformer model in feature extraction and context information integration. However, through effective combination of these two methods, such as TransUnet (R50-ViT) and our DDTransUNet, segmentation performance can be significantly augmented. This fusion approach not only boosts the benefits of the CNN in local feature extraction but also fully leverages the Transformer’s advantages in global information modeling, leading to more precise ultrasound image segmentation.

Our proposed model outperforms TransUnet significantly in terms of performance, primarily attributed to the meticulously crafted dual-branch encoder. This encoder efficiently extracts both global and local feature information from images. Additionally, our introduced CAF module skillfully integrates these two types of information. In feature fusion, convolution-based attention mechanisms (Chen H. et al., 2020) can enhance feature characterization effectively. However, their constrained receptive field size may result in inadequate extraction of global contextual information, thus limiting the ability to accurately capture and integrate global features. Compared to convolution-based attention feature fusion methods, the CAF module offers substantial advantages. Firstly, it effectively captures both macro-structural features and micro-details information. The integrated cross-attention mechanism excels at handing features with complex spatial dependencies, significantly enhancing feature map characterization. Secondly, the meticulously designed attnetion mechanism of the CAF module optimizes computational complexity while managing high efficiency. Compared to existing methods (Zhang et al., 2024), the CAF module excels in multi-branch feature fusion, enabling deep interaction between global and local features, thus improving segmentation accuracy. Its flexibility allows seamless integration into various network, making it suitable for complex visual tasks. The DDTransUNet combines global spatial attention and global channel attention. This combination enables more effective modeling long-range visual dependencies compared to window-based spatial attention methods.

**FIGURE 8:**
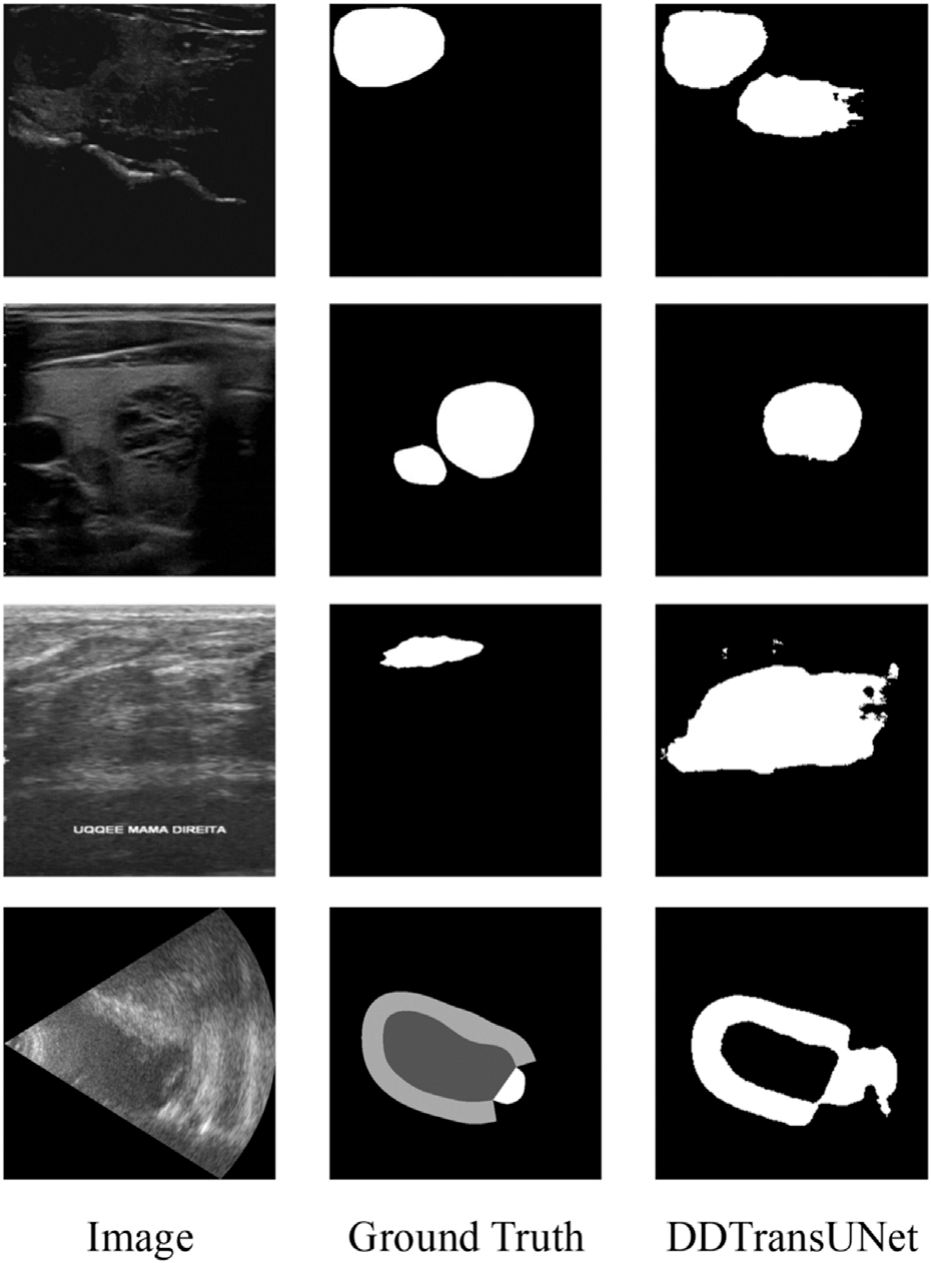
Visualization of the inaccurate segmentation results.

We conduct a comprehensive error analysis to identify and quantify the critical factors influencing the precision of lesion region segmentation in US images. As shown in Figure 8, a set of visual and computational challenges arises when the complexity of the internal tissue structure or the specificity of its characteristics during US imaging leads to an abnormally high shadow density within the region. This phenomenon of elevated shadow density not only intensifies contrast imbalances within the image but also markedly obscures the natural boundary between lesion tissue and the surrounding healthy tissue, thus presenting a substantial challenge for accurate recognition and segmentation.

However, this study still has some limitations. First, the Transformer-based architecture heavily relies on large-scale datasets to capture complex patterns and feature representations, a challenge that is particularly pronounced with small datasets. Due to the absence of inductive bias inherent in CNNs, the performance of the proposed network on small datasets may be somewhat limited. Second, during the current exploratory phase, this study concentrates on three datasets. While this focus yields valuable insights into the characteristics of US images in specific disease states, it somewhat restricts the capacity to capture the extensive range of complex and variable medical scenarios encountered in real-world clinical settings. In clinical practice, individual patient differences, the diversity of disease processes, and the specificity of US responses among various organs and tissues coalesce to form a highly complex and variable US imaging ecosystem. Therefore, a limitation of the current study is the potential oversight of these widespread variability factors, which can profoundly impact the accuracy, sensitivity, and specificity of US diagnosis. To overcome this challenge, future work will focus on integrating self-supervised learning techniques to enable the model to autonomously discover and learn more comprenhensive and rich feature representations, thereby effectively enhancing its performance across datastes of various sizes. To unlock the full potential of ultrasonography in clinical medicine, we plan to expand future research beyond the current linited focus. This will involve creating more comprehensive US imaging datasets that cover a variety of organs, tissues, and patient demographics, including different ages, genders, races, genetic backgrounds, and disease states. By systematically analyzing these diverse datastes, we aim to identify key factors that influence US diagnostic accuracy, ultimately enhancing its applicability across a broader patient population and supporting advancements in precision medicine. Simultaneously, we will prioritize establishing clinical relevance by aligning our research closely with real-world medical needs. This will include developing refined clinical models, analyzing the impact of our findings on patient outcomes and healthcare efficiency, and proposing workflow optimization strategies through system analysis. We will also emphasize practical application by incorporating case studies, conducting multi-center clinical trials, and engaging with a wide range of stakeholders to critically evaluate our research and guide future developments.

## 6 Conclusion

In this paper, we have proposed DDTransUNet, a hybrid network combining Transformer and CNN, designed for the segmentation of ultrasound images. The DDTransUNet is built on a dual-branch encoder and global dual attention mechanism. The dual-branch encoder contains a hierarchical Swin Transformer branch and a CNN branch, facilitating the extraction of both local and global features. The global dual attention consists of Global Spatial Attention (GSA) and Global Channel Attention (GCA), capturing more global contextual information across the spatial and channel dimensions successively. Moreover, we propose a novel Cross Attention Fusion (CAF) module, aiming to amalgamate extensive contextual information and fine-grained feature details through a cross-attention mechanism. Extensive experiments conducted on three different ultrasound image datasets have demonstrated that the proposed DDTransUNet has more superior performance compared to other methods.

## Data Availability

The original contributions presented in the study are included in the article/supplementary material, further inquiries can be directed to the corresponding authors.

## References

[B1] Agarap A. F. (2018). Deep learning using rectified linear units (ReLU). ArXiv abs/1803.08375.

[B2] Ba J. Kiros J. R. Hinton G. E. (2016). Layer normalization. ArXiv abs/1607.06450.

[B3] Bi H. Cai C. Sun J. Jiang Y. Lu G. Shu H. (2023). BPAT-UNet: boundary preserving assembled transformer UNet for ultrasound thyroid nodule segmentation. Comput. Methods Programs Biomed. 238, 107614. 10.1016/j.cmpb.2023.107614 37244233

[B4] Cabria I. Gondra I. (2017). MRI segmentation fusion for brain tumor detection. Inf. Fusion 36, 1–9. 10.1016/j.inffus.2016.10.003

[B5] Cao H. Wang Y. Chen J. Jiang D. Zhang X. Tian Q. (2023). “Swin-unet: unet-like pure transformer for medical image segmentation,” in Computer vision – ECCV 2022 workshops. Editors Karlinsky L. Michaeli T. Nishino K. (Springer Nature Switzerland), 205–218.

[B6] Carion N. Massa F. Synnaeve G. Usunier N. Kirillov A. Zagoruyko S. (2020). “End-to-End object detection with transformers,” in Computer vision – eccv 2020. Editors Vedaldi A. Bischof H. Brox T. Frahm J.-M. (Springer International Publishing), 213–229.

[B7] Chen H. Qi Y. Yin Y. Li T. Liu X. Li X. (2020). MMFNet: a multi-modality MRI fusion network for segmentation of nasopharyngeal carcinoma. Neurocomputing 394, 27–40. 10.1016/j.neucom.2020.02.002

[B8] Chen J. Lu Y. Yu Q. Luo X. Adeli E. Wang Y. (2021). TransUNet: transformers make strong encoders for medical image segmentation. *ArXiv* abs/2102.04306.

[B9] Chen J. You H. Li K. (2020). A review of thyroid gland segmentation and thyroid nodule segmentation methods for medical ultrasound images. Comput. Methods Programs Biomed. 185, 105329. 10.1016/j.cmpb.2020.105329 31955006

[B10] Cheng J. Ye J. Deng Z. Chen J. Li T. Wang H. (2023). Sam-med2d. *arXiv preprint arXiv:2308.16184* .

[B11] Çiçek Ö. Abdulkadir A. Lienkamp S. S. Brox T. Ronneberger O. (2016). “3D U-net: learning dense volumetric segmentation from sparse annotation,” in Medical image computing and computer-assisted intervention – miccai 2016. Editors Ourselin S. Joskowicz L. Sabuncu M. R. Unal G. Wells W. (Springer International Publishing), 424–432.

[B12] Devlin J. Chang M.-W. Lee K. Toutanova K. (2019). “BERT: pre-training of deep bidirectional transformers for language understanding,” in North American Chapter of the Association for computational linguistics.

[B13] Ding M. Xiao B. Codella N. Luo P. Wang J. Yuan L. (2022). “DaViT: dual attention vision transformers,” in Computer vision – eccv. Editors Avidan S. Brostow G. Cissé M. Farinella G. M. Hassner T. (Springer Nature Switzerland), 74–92.

[B14] Domingues I. Pereira G. Martins P. Duarte H. Santos J. Abreu P. H. (2020). Using deep learning techniques in medical imaging: a systematic review of applications on CT and PET. Artif. Intell. Rev. 53 (6), 4093–4160. 10.1007/s10462-019-09788-3

[B15] Dosovitskiy A. Beyer L. Kolesnikov A. Weissenborn D. Zhai X. Unterthiner T. (2020). An image is worth 16x16 words: transformers for image recognition at scale. *ArXiv* abs/2010.11929.

[B16] Drukker L. Noble J. A. Papageorghiou A. T. (2020). Introduction to artificial intelligence in ultrasound imaging in obstetrics and gynecology. Ultrasound Obstetr. Gynecol. 56 (4), 498–505. 10.1002/uog.22122 PMC7702141 32530098

[B17] Gao Y. Zhou M. Metaxas D. N. (2021). “UTNet: a hybrid transformer architecture for medical image segmentation,” in Medical image computing and computer assisted intervention – miccai 2021. Editors de Bruijne M. Cattin P. C. Cotin S. Padoy N. Speidel S. Zheng Y. (Springer International Publishing), 61–71.

[B18] Gómez-Flores W. Gregorio-Calas M. J. Coelho de Albuquerque Pereira W. (2024). BUS-BRA: a breast ultrasound dataset for assessing computer-aided diagnosis systems. Med. Phys. 51 (4), 3110–3123. 10.1002/mp.16812 37937827

[B19] Gong H. Chen G. Wang R. Xie X. Mao M. Yu Y. (2021). “Multi-task learning for thyroid nodule segmentation with thyroid region prior,” in 2021 IEEE 18th International Symposium on Biomedical Imaging (ISBI), Nice, France, 13-16 April 2021, 257–261.

[B20] Guo J. Zhou H.-Y. Wang L. Yu Y. (2023). “UNet-2022: exploring dynamics in non-isomorphic architecture,” in Medical imaging and computer-aided diagnosis. Editors Su R. Zhang Y. Liu H. F Frangi A. (Springer Nature Singapore), 465–476.

[B21] Hatamizadeh A. Nath V. Tang Y. Yang D. Roth H. R. Xu D. (2022). “Swin UNETR: Swin transformers for semantic segmentation of brain tumors in MRI images,” in Brainlesion: glioma, multiple sclerosis, stroke and traumatic brain injuries. Editors Crimi, A. Bakas S. (Springer International Publishing), 272–284.

[B22] Hatamizadeh A. Yang D. Roth H. R. Xu D. (2021). “UNETR: transformers for 3D medical image segmentation,” in 2022 IEEE/CVF Winter Conference on Applications of Computer Vision (WACV), Waikoloa, HI, USA, 03-08 January 2022, 1748–1758.

[B23] Huang Q. Zhang F. Li X. (2018). Machine learning in ultrasound computer-aided diagnostic systems: a survey. BioMed Res. Int. 2018, 5137904. 10.1155/2018/5137904 29687000 PMC5857346

[B24] Huang X. Deng Z. Li D. Yuan X. Fu Y. (2023). MISSFormer: an effective transformer for 2D medical image segmentation. IEEE Trans. Med. Imaging 42 (5), 1484–1494. 10.1109/TMI.2022.3230943 37015444

[B25] Ioffe S. Szegedy C. (2015). “Batch normalization: accelerating deep network training by reducing internal covariate shift,” in Proceedings of the 32nd international conference on machine learning. Editors Francis, B. David B. (Lille, France: Proceedings of Machine Learning Research, PMLR).

[B26] Jha D. Smedsrud P. H. Riegler M. A. Johansen D. Lange T. D. Halvorsen P. (2019). “ResUNet++: an advanced architecture for medical image segmentation,” in 2019 IEEE International Symposium on Multimedia (ISM), San Diego, CA, USA, 09-11 December 2019, 225–2255.

[B27] Jiang Z. Salcudean S. E. Navab N. (2023). Robotic ultrasound imaging: state-of-the-art and future perspectives. Med. Image Anal. 89, 102878. 10.1016/j.media.2023.102878 37541100

[B28] Kirillov A. Mintun E. Ravi N. Mao H. Rolland C. Gustafson L. (2023). “Segment anything,” in 2023 IEEE/CVF international conference on computer vision (ICCV), 3992–4003.

[B29] Le N. Q. K. (2023). Predicting emerging drug interactions using GNNs. Nat. Comput. Sci. 3 (12), 1007–1008. 10.1038/s43588-023-00555-7 38177735

[B30] Leclerc S. Smistad E. Pedrosa J. Østvik A. Cervenansky F. Espinosa F. (2019). Deep learning for segmentation using an open large-scale dataset in 2D echocardiography. IEEE Trans. Med. Imaging 38 (9), 2198–2210. 10.1109/TMI.2019.2900516 30802851

[B31] Li J. Chen J. Tang Y. Wang C. Landman B. A. Zhou S. K. (2023). Transforming medical imaging with Transformers? A comparative review of key properties, current progresses, and future perspectives. Med. Image Anal. 85, 102762. 10.1016/j.media.2023.102762 36738650 PMC10010286

[B32] Li X. Pang S. Zhang R. Zhu J. Fu X. Tian Y. (2023). ATTransUNet: an enhanced hybrid transformer architecture for ultrasound and histopathology image segmentation. Comput. Biol. Med. 152, 106365. 10.1016/j.compbiomed.2022.106365 36516577

[B33] Liu X. Peng H. Zheng N. Yang Y. Hu H. Yuan Y. (2023). “EfficientViT: memory efficient vision transformer with cascaded group attention,” in 2023 IEEE/CVF Conference on Computer Vision and Pattern Recognition (CVPR), Vancouver, BC, Canada, 17-24 June 2023, 14420–14430.

[B34] Liu Z. Lin Y. Cao Y. Hu H. Wei Y. Zhang Z. (2021). “Swin transformer: hierarchical vision transformer using shifted windows,” in 2021 IEEE/CVF International Conference on Computer Vision (ICCV), 9992–10002.

[B35] Ma J. He Y. Li F. Han L. You C. Wang B. (2024). Segment anything in medical images. Nat. Commun. 15 (1), 654. 10.1038/s41467-024-44824-z 38253604 PMC10803759

[B36] Milletari F. Navab N. Ahmadi S. A. (2016). “V-net: fully convolutional neural networks for volumetric medical image segmentation,” in 2016 Fourth International Conference on 3D Vision (3DV), Stanford, CA, USA, 25-28 October 2016, 565–571.

[B37] Naseer M. Ranasinghe K. Khan S. H. Hayat M. Khan F. S. Yang M.-H. (2021). “Intriguing properties of vision transformers,” in Neural information processing systems.

[B38] Niu D. Wang X. Han X. Lian L. Herzig R. Darrell T. (2023). Unsupervised universal image segmentation. *ArXiv* abs/2312.17243.

[B39] Oktay O. Schlemper J. Folgoc L. L. Lee M. J. Heinrich M. P. Misawa K. (2018). Attention U-net: learning where to look for the pancreas. *ArXiv* abs/1804.03999.

[B40] Peng Y. Sonka M. Chen D. Z. (2023). U-net v2: rethinking the skip connections of U-net for medical image segmentation. *ArXiv* abs/2311.17791.

[B41] Qi H. Wang W. Shi Y. Wang X. (2024). AD-DUNet: a dual-branch encoder approach by combining axial Transformer with cascaded dilated convolutions for liver and hepatic tumor segmentation. Biomed. Signal Process. Control 95, 106397. 10.1016/j.bspc.2024.106397

[B42] Ronneberger O. Fischer P. Brox T. (2015). “U-net: convolutional networks for biomedical image segmentation,” in Medical image computing and computer-assisted intervention – miccai 2015. Editors Navab N. Hornegger J. Wells W. M. Frangi A. F. (Springer International Publishing), 234–241.

[B43] Shaker A. M. Maaz M. Rasheed H. A. Khan S. H. Yang M. Khan F. S. (2022). UNETR++: delving into efficient and accurate 3D medical image segmentation. *ArXiv* abs/2212.04497. 10.1109/TMI.2024.3398728 38722726

[B44] Shamshad F. Khan S. Zamir S. W. Khan M. H. Hayat M. Khan F. S. (2023). Transformers in medical imaging: a survey. Med. Image Anal. 88, 102802. 10.1016/j.media.2023.102802 37315483

[B45] Sloun R. J. G. v. Cohen R. Eldar Y. C. (2020). Deep learning in ultrasound imaging. Proc. IEEE 108 (1), 11–29. 10.1109/JPROC.2019.2932116

[B46] Taud H. Mas J. F. (2018). “Multilayer Perceptron (MLP),” in Geomatic approaches for modeling land change scenarios. Editors Camacho Olmedo M. T. Paegelow M. Mas J.-F. Escobar F. (Cham: Springer International Publishing), 451–455.

[B47] Touvron H. Cord M. Douze M. Massa F. Sablayrolles A. Jegou H. (2021). “Training data-efficient image transformers and distillation through attention,” in Proceedings of the 38th international conference on machine learning. Editors Marina, M. Tong Z. (Proceedings of Machine Learning Research: PMLR).

[B48] Tran T.-O. Vo T. H. Le N. Q. K. (2023). Omics-based deep learning approaches for lung cancer decision-making and therapeutics development. Briefings Funct. Genomics 23 (3), 181–192. 10.1093/bfgp/elad031 37519050

[B49] Tuli S. Dasgupta I. Grant E. Griffiths T. L. (2021). Are convolutional neural networks or transformers more like human vision? ArXiv abs/2105.07197.

[B50] Valanarasu J. M. J. Patel V. M. (2022). “UNeXt: MLP-based rapid medical image segmentation network,” in Medical image computing and computer assisted intervention – miccai 2022. Editors Wang L. Dou Q. Fletcher P. T. Speidel S. Li S. (Springer Nature Switzerland), 23–33.

[B51] Vaswani A. Shazeer N. M. Parmar N. Uszkoreit J. Jones L. Gomez A. N. (2017). “Attention is all you need,” in Neural information processing systems.

[B52] Wang H. Xie S. Lin L. Iwamoto Y. Han X. H. Chen Y. W. (2022). “Mixed transformer U-net for medical image segmentation,” in ICASSP 2022 - 2022 IEEE International Conference on Acoustics, Speech and Signal Processing (ICASSP), Singapore, 23-27 May 2022, 2390–2394.

[B53] Wang W. Xie E. Li X. Fan D. P. Song K. Liang D. (2021). “Pyramid vision transformer: a versatile backbone for dense prediction without convolutions,” in 2021 IEEE/CVF International Conference on Computer Vision (ICCV), Montreal, QC, Canada, 10-17 October 2021, 548–558.

[B54] Xian M. Zhang Y. Cheng H. D. Xu F. Zhang B. Ding J. (2018). Automatic breast ultrasound image segmentation: a survey. Pattern Recognit. 79, 340–355. 10.1016/j.patcog.2018.02.012

[B55] Xiao X. Lian S. Luo Z. Li S. (2018). “Weighted res-UNet for high-quality retina vessel segmentation,” in 2018 9th International Conference on Information Technology in Medicine and Education (ITME), Hangzhou, China, 19-21 October 2018, 327–331.

[B56] Xu G. Zhang X. He X. Wu X. (2024). LeViT-UNet: make faster encoders with transformer for medical image segmentation. Springer Nature Singapore, 42–53.

[B57] Ye Y. Xie Y. Zhang J. Chen Z. Xia Y. (2023). UniSeg: a prompt-driven universal segmentation model as well as A strong representation learner. Springer Nature Switzerland, 508–518.

[B58] Yuan L. Chen Y. Wang T. Yu W. Shi Y. Jiang Z. (2021). “Tokens-to-Token ViT: training vision transformers from scratch on ImageNet,” in 2021 IEEE/CVF International Conference on Computer Vision (ICCV), 538–547.

[B59] Zhang H. Lian J. Yi Z. Wu R. Lu X. Ma P. (2024). HAU-Net: hybrid CNN-transformer for breast ultrasound image segmentation. Biomed. Signal Process. Control 87, 105427. 10.1016/j.bspc.2023.105427

[B60] Zhou H. Y. Guo J. Zhang Y. Han X. Yu L. Wang L. (2023). nnFormer: volumetric medical image segmentation via a 3D transformer. IEEE Trans. Image Process. 32, 4036–4045. 10.1109/TIP.2023.3293771 37440404

[B61] Zhou Z. Rahman Siddiquee M. M. Tajbakhsh N. Liang J. (2018). “UNet++: a nested U-net architecture for medical image segmentation,” in Deep learning in medical image analysis and multimodal learning for clinical decision support. Editors Stoyanov D. Taylor Z. Carneiro G. Syeda-Mahmood T. Martel A. Maier-Hein L. (Springer International Publishing), 3–11. 10.1007/978-3-030-00889-5_1 PMC7329239 32613207

[B62] Zhu Z. He X. Qi G. Li Y. Cong B. Liu Y. (2023). Brain tumor segmentation based on the fusion of deep semantics and edge information in multimodal MRI. Inf. Fusion 91, 376–387. 10.1016/j.inffus.2022.10.022

[B63] Zhu Z. Wang Z. Qi G. Mazur N. Yang P. Liu Y. (2024). Brain tumor segmentation in MRI with multi-modality spatial information enhancement and boundary shape correction. Pattern Recognit. 153, 110553. 10.1016/j.patcog.2024.110553

